# *Cordyceps cicadae* NTTU 868 Mycelia Fermented with Deep Ocean Water Minerals Prevents D-Galactose-Induced Memory Deficits by Inhibiting Oxidative Inflammatory Factors and Aging-Related Risk Factors

**DOI:** 10.3390/nu15081968

**Published:** 2023-04-19

**Authors:** Ching-Yu Chang, Pei-Xin Yang, Tsai-Luen Yu, Chun-Lin Lee

**Affiliations:** 1 Marine Industry and Engineer Research Center, National Academy of Marine Research, Kaohsiung 806614, Taiwan; 2 Department of Life Science, National Taitung University, 369, Section 2, University Rd., Taitung 95092, Taiwan

**Keywords:** deep ocean water, *Cordyceps cicadae*, minerals, polysaccharide, N(6)-(2-Hydroxyethyl) adenosine

## Abstract

*Cordyceps cicadae*, a medicinal fungus that is abundant in bioactive compounds such as N6-(2-hydroxyethyl)-adenosine (HEA) and polysaccharides, possesses remarkable anti-inflammatory, antioxidant, and nerve damage recovery properties. Deep ocean water (DOW) contains minerals that can be absorbed and transformed into organic forms by fungi fermentation. Recent studies have shown that culturing *C. cicadae* in DOW can enhance its therapeutic benefits by increasing the levels of bioactive compounds and minerals’ bioavailibility. In this study, we investigated the effects of DOW-cultured *C. cicadae* (DCC) on brain damage and memory impairment induced by D-galactose in rats. Our results indicate that DCC and its metabolite HEA can improve memory ability and exhibit potent antioxidant activity and free radical scavenging in D-galactose-induced aging rats (*p* < 0.05). Additionally, DCC can mitigate the expression of inflammatory factors, such as tumor necrosis factor-alpha (TNF-α), interleukin-6 (IL-6), interleukin-1β (IL-1β), cyclooxygenase-2 (COX-2), and inducible nitric oxide synthase (iNOS), thereby preventing brain aging. Furthermore, DCC showed a significant decrease in the expression of the aging-related proteins glial fibrillary acidic protein (GFAP) and presenilin 1 (PS1). By reducing brain oxidation and aging-related factors, DOW-cultured *C. cicadae* demonstrate enhanced anti-inflammatory, antioxidant, and neuroprotective effects, making it a promising therapeutic agent for preventing and treating age-related brain damage and cognitive impairment.

## 1. Introduction

Aging is characterized by a progressive decline in physiological functions over time. The etiology of aging is multifactorial, including genetic, hormonal, free radical, and autoimmune factors. The free radical theory of aging has been widely supported by previous studies, as the accumulation of oxidative stress is closely related to aging. The brain is particularly vulnerable to the detrimental effects of oxidative stress, which can ultimately result in impaired cognitive function and the development of dementia [1]. Previous studies have demonstrated that intraperitoneal (i.p.) administration of high doses of D-galactose can result in dysfunction of the mitochondrial electron transport chain, leading to elevated levels of advanced glycation end products (AGEs) and superoxide radicals. This, in turn, triggers the generation of reactive oxygen species (ROS) and subsequent oxidative stress. Oxidative stress is known to promote the expression of inflammatory cytokine markers, including cyclooxygenase-2 (COX-2), inducible nitric oxide synthase (iNOS), tumor necrosis factor-alpha (TNF-α), interleukin-1β (IL-1β), interleukin-6 (IL-6), and nuclear factor kappa-light-chain-enhancer of activated B (NF-κB). As a consequence, mitochondrial function is compromised and the activity of the JNK pathway is heightened, ultimately leading to apoptosis and nerve damage in the brain [2].

Deep ocean water (DOW) is seawater found below the thermocline of the ocean, typically at depths greater than 200 m. This water is characterized by its low temperature, cleanliness, and high concentration of minerals and salt nutrients. Past studies have demonstrated the potential for DOW to prevent obesity [3], cardiovascular disease [4], and anti-atherosclerosis [5] developments. In a previous study, it was discovered that the use of DOW in the fermentation process of *Cordyceps militaris* resulted in enhanced efficacious ingredients, including adenosine and cordycepin. These compounds were found to reduce the expression of fibrosis-related factors by inhibiting thioacetamide (TAA)-induced inflammatory factor expression and increasing PPAR-γ activity [3]. Animal experiments were conducted to investigate the effects of DOW on liver fibrosis using *Antrodia camphorata*-fermented products cultured with DOW or ultrapure water. The group fed with DOW-cultured products showed a significantly greater inhibitory effect on lipid peroxidation, ROS, iNOS, and TNF-α compared to the group fed with *Antrodia camphorata* fermented products cultured with ultrapure water [6].

*Cordyceps cicadae* is a traditional Chinese medicinal fungus that can be cultured in a silkworm, cereal, and potato glucose medium. Previous studies have identified several effective components of *Cordyceps cicadae*, including adenosine, N6-(2-hydroxyethyl)-adenosine (HEA), polysaccharides, and ergosterol, which possess antioxidant, anti-inflammatory, and antiaging properties. In an experiment using LPS-induced inflammation in RAW 264.7 macrophages, treatment with the methanol extract of *C. cicadae* effectively reduced the inflammatory factors COX-2, Prostaglandin E2 (PGE2), IL-1β, and TNF-α. Furthermore, HEA extracted from *C. cicadae* inhibited the NF-κB signaling pathway to achieve anti-inflammatory effects [7]. Prior research has demonstrated that in H_2_O_2_-induced oxidative damage experiments in PC12 cells, HEA improves cell viability and reduces LDH release, MMP deficiency, and ROS generation caused by H_2_O_2_ toxicity. HEA also enhances the activity of antioxidant enzymes and suppresses the production of inflammatory factors by inhibiting lipid peroxidation [8]. A previous study used DOW-fermented *C. cicadae* (DCC) mycelium to induce Alzheimer’s disease by intracerebral injection of amyloid β-protein 40 in rats. After 28 days of testing, the rats showed improvements in memory and learning, as well as brain damage. DCC can significantly improve the absorption of magnesium ions in the brain by increasing the expression of magnesium transporters, thereby achieving the effect of improving Alzheimer’s disease [9].

Based on the above, it is evident that *C. cicadae* possess potential anti-inflammatory and antioxidant properties. Co-culturing with DOW can enhance its preventive and ameliorating effects on brain injury. However, oxidative stress is a critical factor in brain aging and cognitive decline. This study aims to investigate the efficacy of using *C. cicadae* mycelium products cultured in DOW to prevent D-galactose-induced brain injury and memory impairment in rats, with the aim of developing fermentation products that can enhance brain neuroprotection.

## 2. Materials and Methods

### 2.1. Chemicals 

Potato dextrose agar (PDA) and potato dextrose broth (PDB) were purchased from Difco Co. (Detroit, MI, USA). D-galactose and N(6)-(2-Hydroxyethyl) adenosine (HEA) (>95% purity) were purchased from Sigma Chemical Co. (St Louis, MO, USA). Mouse TNF-α protein (50349-MNAE), mouse IL-6 protein (50349-MNAE), mouse IL-1β protein (50349-MNAE), and rat iNOS protein (Q06518) were purchased from SinoBiological Inc. (North Wales, PA, USA). The mouse anti-rat IL-6 monoclonal antibody (sc-57315) and the mouse anti-human IL-1β monoclonal antibody (sc-32294) were purchased from Santa Cruz Biotechnology, Inc. (Dallas, TX, USA). The rabbit iNOS antibody (FNab04325), the rabbit anti-rat COX-2 polyclonal antibody (RPD284Ra01), and the rat COX-2 protein (RPD284Ra01) were purchased from Cloud-Clone Corp. (Wuhan, China).The rRabbit anti-mouse TNF-α polyclonal antibody (AB2148P) was purchased from the EMD Millipore Corporation (Temecula, CA, USA). Peroxidase conjugated antibodies (31460) were purchased from Pierce Biotechnology (Rockford, IL, USA).

### 2.2. The Source of DOW

The concentrated deep ocean water (DOW) utilized in this study was obtained from the astern Taiwan Deep Seawater Innovation Research Center located in Taitung, Taiwan. The DOW was extracted from a depth of 670 m in the Pacific Ocean near eastern Taiwan. In a previous study conducted by our research group, it was determined that the DOW containing 20.65 mg/L Mg^2+^ would be classified as double-strength DOW. For this present study, a 15-fold DOW was prepared by diluting concentrated DOW (82640 mg/L Mg^2+^) with ultrapure water (UPW). The 15X DOW contained 309.75 mg/L Mg^2+^, 24.08 mg/L Na^+^, 36.56 mg/L K^+^, 104.95 mg/L Ca^2+^, 0.0012 mg/L Zn^2+^, and 0.0024 mg/L Cu^2+^. These concentrations of trace elements and minerals were analyzed and reported in a study conducted by Wu and Lee in 2021 [9].

### 2.3. Sample Preparation

The UPW medium with 75.25 mg/L of Mg^2+^ concentration was made by dissolving a potato dextrose broth (PDB) medium in ultrapure water. The DOW medium with 385 mg/L of Mg^2+^ concentration was made by dissolving a PDB medium in 15X-DOW with 309.75 mg/L of Mg^2+^. The MgCl_2_ medium with 385 mg/L of Mg^2+^ concentration was made by dissolving a PDB medium in a MgCl_2_ solution with 309.75 mg/L of Mg^2+^.

The *C. cicadae* NTTU 868 fermentation product was obtained from a 1.5 L submerged fermentation. *C. cicadae* NTTU 868 was seed cultured in PDB at 23 °C for 3 days, then inoculated (10%) into the UPW medium, DOW medium, or MgCl_2_ medium and cultured at 23 °C for 10 days. After fermentation, *C. cicadae* fermentation products cultured with UPW (UCC), DOW (DCC), and magnesium chloride solution (MgCC) were obtained, respectively.

To obtain the mycelium, the fermentation product was centrifuged at 10,000× *g* for 15 min at +4 °C. The mycelium was dried at 50 °C, ground into powder, and stored at room temperature. The supernatant (culture fluid) obtained after centrifugation was stored at +4 °C. All submerged fermentation products (including mycelium and culture fluid) were homogenized to prepare animal test samples, the dry weight of the mycelium was calculated proportionally, and the weight per milliliter of mycelium was determined before preparing and administering the test samples.

Previous experiments have shown that different *C. cicadae* NTTU 868 fermentation products have varying levels of HEA, polysaccharides, and intracellular magnesium content. UCC had 0.88 mg/L of HEA, 648 mg/L of polysaccharides and 10.1 mg/L of intracellular magnesium content. DCC had 1.04 mg/L of HEA, 591 mg/L of polysaccharides and 161 mg/L of intracellular magnesium content. MgCC had 1.32 mg/L of HEA, 628 mg/L of polysaccharides and 132 mg/L of intracellular magnesium content [9]. The main difference between DCC and UCC was the higher accumulation of intracellular and extracellular deep seawater minerals in DCC. MgCC had the same amount of magnesium ion (Mg^2+^) as DCC but a lower intracellular magnesium ion (Mg^2+^) content than DCC.

### 2.4. Animals Grouping and Experiment Schedule 

Individually housed male Sprague Dawley (SD) rats, aged 8 weeks, were purchased from the BioLasco Co. in Taipei, Taiwan. They were provided with free access to standard laboratory chow (Ralston Purina, St. Louis, MO, USA) and water, and were kept in a temperature-controlled room at 24 °C with a 12 h light–dark cycle (lights on at 6:00). The rats were given food ad libitum throughout the experiment. To form the control group, 42 rats were randomly divided into 6 groups and fed a standard diet (NOR; 4.5% fat, 3.34 kcal/g). 

During the experiment, all groups except for the control group (NOR group) were subcutaneously injected with 500 mg/kg b.w. of D-galactose per day and fed with the test substance for 56 days. The test substance was *C. cicadae* after 10 days of liquid fermentation; the mycelium was obtained, dried at 50 °C and ground into powder. An adult (60 kg) was suggested to consume 250 mL of *C. cicadae* fermented product. The DCC group was fed with the total *C. cicadae* product of DOW liquid state fermentation (26 mL/kg b.w./day) every day, and the MgCC group was fed with the DCC group with the same magnesium ion (Mg^2+^) content as the DCC group. The *C. cicadae* total product of liquid state fermentation (26 mL/kg b.w./day). The UCC group was fed with the total product of *C. cicadae* (26 mL/kg b.w./day) from UPW fermentation every day, and the HEA group was fed with the functional ingredient standard product of the same HEA concentration as the DCC group every day. Rat body weights were recorded weekly.

After 9 weeks of feeding, the rats were humanely euthanized for tissue collection. The collected mouse serum was immediately stored at −80 °C until further analysis. The brain tissue was dissected into hippocampus and cortex, and the cortex was further divided into left and right hemispheres, and then stored at −80 °C until protein analysis. This study was conducted in accordance with the ethical guidelines established by the Animal Care and Research Ethics Committee of National Taitung University and was approved by the same committee.

### 2.5. Animal Learning and Memory Tests

#### 2.5.1. Morris Water Maze Task 

The water maze test was pioneered by the British psychologist Morris in 1981 to study the learning, memory, and spatial cognition abilities of rats with damaged brain regions such as the hippocampus. In this test, the animals’ abilities can be evaluated by observing and recording their swimming time for searching the resting platform under the water after entering the water. The test apparatus is a circular pool with a diameter of 168 cm and a water depth of 40 cm, and it contains a movable resting platform that is 10 cm in diameter and 5 cm above the water surface. The swimming pool is divided into quadrants I, II, III, and IV, with 5 starting points. The rest platform is located in the center of the third quadrant to avoid animals seeing the platform and affecting the experiment. During the experiment, a video camera (DCM-LX5, Panasonic Co, Osaka, Japan) was set up above the center of the swimming pool to record the time when the experimental animals were looking for the rest platform and swimming lane [10].

#### 2.5.2. Passive Avoidance Task

The passive avoidance test assesses the memory and learning abilities of rats. It consists of two equally sized rooms, one dark and one light, separated by a movable door. A metal rod connected to an electric current supply is present in the dark room to deliver an electric shock. Rats are placed in the bright room and allowed to acclimate before the door is opened to enter the dark room. The time spent by the rats in the bright room is recorded, and they receive an electric shock upon entering the dark room. The test is repeated thrice daily for three consecutive days [11].

### 2.6. Preparation of Brains 

The method for brain processing was adapted from a 2021 study by Wu and Lee. The cerebral cortex and hippocampus were carefully isolated from the whole brains on ice and any blood was removed. The isolated tissues were then snap-frozen using liquid nitrogen and stored at −80 °C until further use. For protein extraction in preparation for immunoblotting, 100 mg of tissue was homogenized in 1.0 mL of lysis buffer containing various reagents, including Triton X-100, Tris, NaCl, NaF, SDS, deoxycholate, EDTA, EGTA, and Na3VO4. The homogenate was then briefly sonicated for 10 s before being centrifuged at 100,000× *g* for 30 min. The resulting supernatant was utilized for the immunoblotting assay.

### 2.7. Enzyme-Linked Immunosorbent Assay 

The protein content of the samples was analyzed with a commercially available BCA protein assay kit (23225, Thermo Fisher Scientific Inc., Rockford, IL, USA). The protein expression analysis steps are as follows. To perform the assay, 100 μL of either the sample or a protein standard (TNF-α, IL-6, IL-1β, COX-2, iNOS) was added to each well of a 96-well plate and incubated at 37 °C for 90 min. Next, the samples or protein standards were removed, and 100 μL of biotinylated detection antibody was added to each well. The plate was then incubated at 37 °C for 60 min. After removing the sample and washing the plate, 100 μL of HRP conjugate was added to each well and the plate was incubated at 37 °C for 30 min. Following the removal of the sample and further washing of the plate, 90 μL of the subtract reagent was added to each well and the plate was incubated at 37 °C for 15 min. Finally, 50 μL of 2 N sulfuric acid was added to the plate to terminate the reaction, and the absorbance was measured at 450 nm using a full-spectrum microplate analyzer (51119300, MultiskanTW Go, Thermo Fisher Scientific Inc., Waltham, MA, USA).

GPx, GRd, and SOD activities were analyzed with commercial kits (Randox Laboratories Ltd., Antrim, UK). Presenilin1 and GFAP protein content were analyzed with commercially available ELISA kits (Wuhan Fine Biotech Co., Ltd., Wuhan, China).

### 2.8. Statistical Analysis 

Data are presented as mean ± standard deviation. Analysis of variance with Duncan’s test and the one-way ANOVA test was determined using SPSS version 12.0 software (SPSS Institute, Inc., Chicago, IL, USA). Differences at *p* < 0.05 were considered statistically significant.

## 3. Results

### 3.1. C. cicadae Products Fermented with Ion Water Improve the Learning and Memory Abilities of Aging Rats Induced by DG

**Figure 1:**
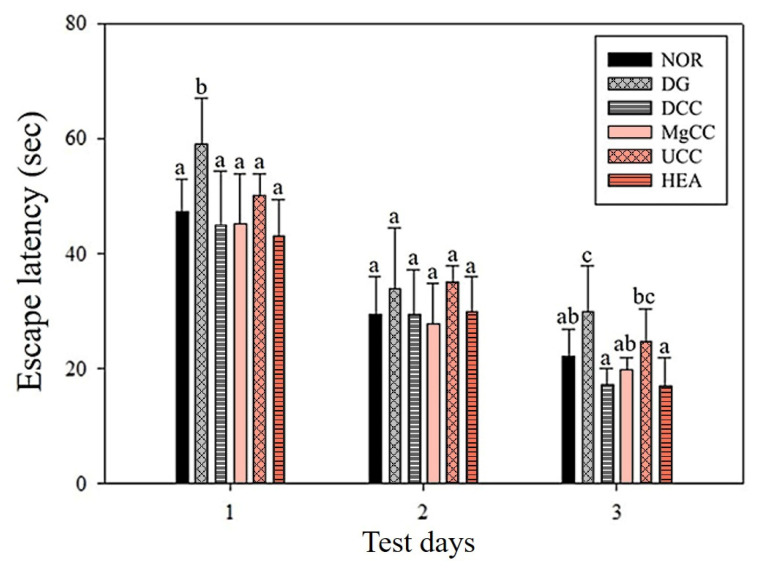
Effects of *C. cicadae* NTTU 868 fermentation products cultured with various water on the platform searching time of DG-induced aging rats in the reference memory test. Animals, with the exception of the NOR group, were injected daily with D-galactose (500 mg/kg b.w./day). The DCC group received daily administration of *C. cicadae* fermentation product cultured with DOW (26 mL/kg b.w./day), while the MgCC group received *C. cicadae* fermentation product cultured with MgCl_2_ solution (26 mL/kg b.w./day). The UCC group was administered *C. cicadae* fermentation product cultured with UPW (26 mL/kg b.w./day), while the HEA group was administered HEA (0.298 µL/kg b.w./day) daily. The data are presented as mean ± standard deviation (n = 7). Mean values within each group with different lowercase letters are significantly different (*p* < 0.05).

**Figure 2:**
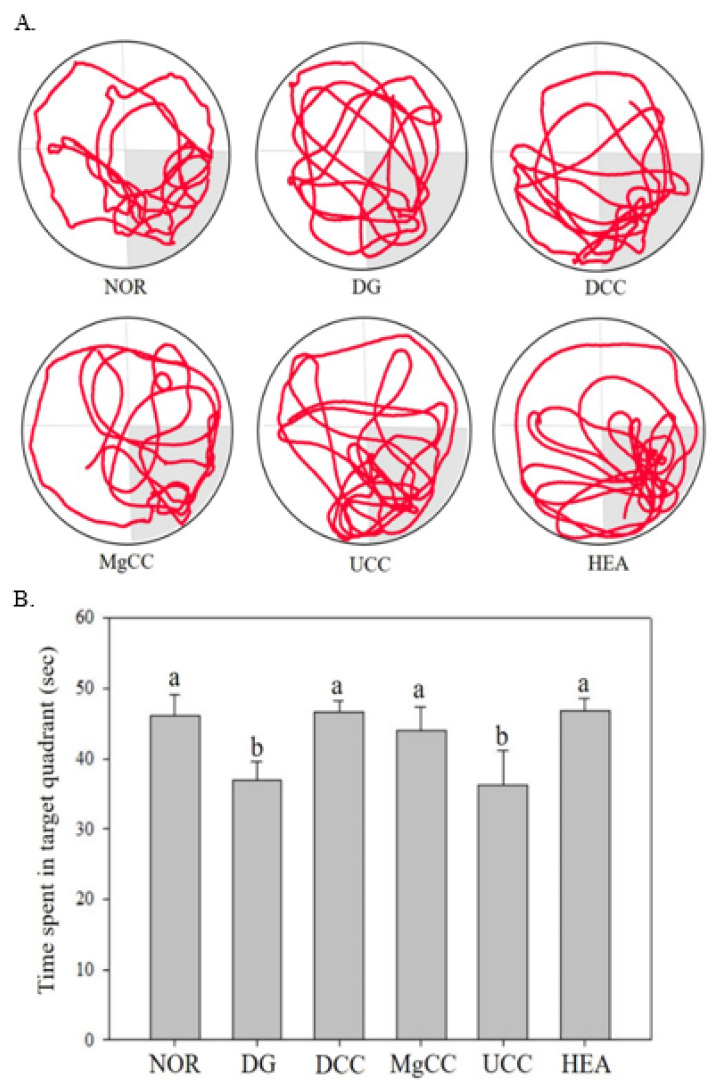
Effects of *C. cicadae* NTTU 868 fermentation products cultured with various water on the platform searching time of DG-induced aging rats in spatial probe test (**A**) Swimming pathway (**B**) Time spent in the target quadrant. Animals, with the exception of the NOR group, were injected daily with D-galactose (500 mg/kg b.w./day). The DCC group received daily administration of *C. cicadae* fermentation product cultured with DOW (26 mL/kg b.w./day), while the MgCC group received *C. cicadae* fermentation product cultured with MgCl_2_ solution (26 mL/kg b.w./day). The UCC group was administered *C. cicadae* fermentation product cultured with UPW (26 mL/kg b.w./day), while the HEA group was administered HEA (0.298 µL/kg b.w./day) daily. The data are presented as mean ± standard deviation (n = 7). Mean values within each group with different lowercase letters are significantly different (*p* < 0.05).

**Figure 3:**
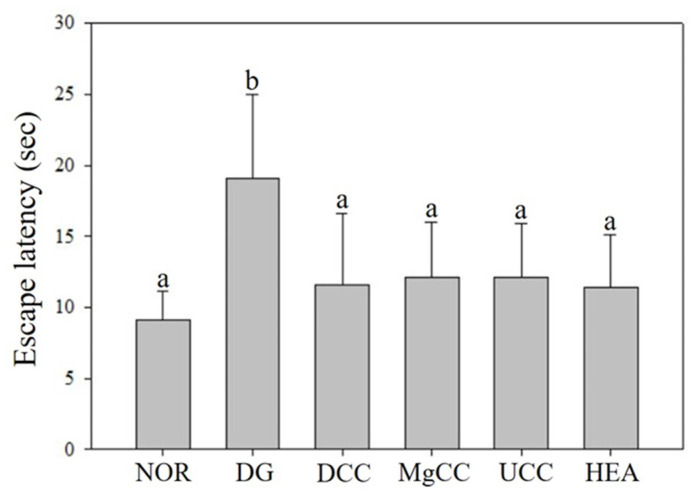
Effects of *C. cicadae* NTTU 868 fermentation products cultured with various water on the platform searching time of DG-induced aging rats in working memory test. Animals, with the exception of the NOR group, were injected daily with D-galactose (500 mg/kg b.w./day). The DCC group received daily administration of *C. cicadae* fermentation product cultured with DOW (26 mL/kg b.w./day), while the MgCC group received *C. cicadae* fermentation product cultured with MgCl_2_ solution (26 mL/kg b.w./day). The UCC group was administered *C. cicadae* fermentation product cultured with UPW (26 mL/kg b.w./day), while the HEA group was administered HEA (0.298 µL/kg b.w./day) daily. The data are presented as mean ± standard deviation (n = 7). Mean values within each group with different lowercase letters are significantly different (*p* < 0.05).

**Figure 4:**
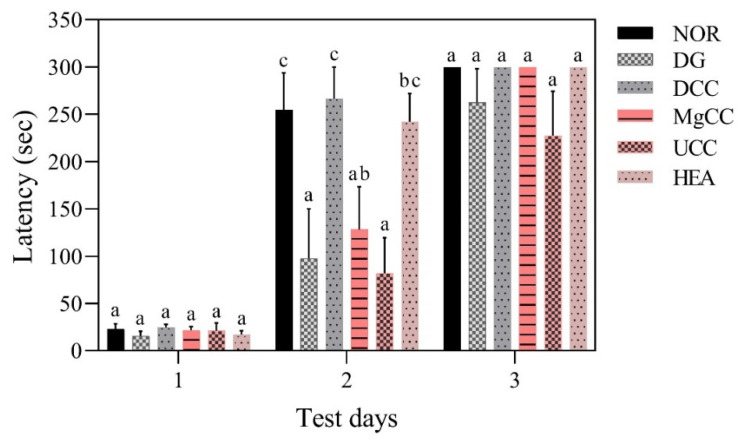
Effects of *C. cicadae* NTTU 868 fermentation products cultured with various water on the latency time of DG-induced aging rats in passive avoidance test. Animals, with the exception of the NOR group, were injected daily with D-galactose (500 mg/kg b.w./day). The DCC group received daily administration of *C. cicadae* fermentation product cultured with DOW (26 mL/kg b.w./day), while the MgCC group received *C. cicadae* fermentation product cultured with MgCl_2_ solution (26 mL/kg b.w./day). The UCC group was administered *C. cicadae* fermentation product cultured with UPW (26 mL/kg b.w./day), while the HEA group was administered HEA (0.298 µL/kg b.w./day) daily. The data are presented as mean ± standard deviation (n = 7). Mean values within each group with different lowercase letters are significantly different (*p* < 0.05).

The reference memory test is an important evaluation index for memory and learning in the water maze. As depicted in Figure 1, the test results on the third day revealed significant differences in memory ability among the animal groups. Specifically, the NOR, DCC, MgCC, and HEA groups showed a shorter time to find the target platform compared to the DG group (*p* < 0.05). In the intergroup comparison of the experimental group, the DCC group and the HEA group had significantly lower swimming times than the UCC group on day 3 (*p* < 0.05). After the reference memory test, the platform was removed to conduct the spatial detection test. The swimming path was analyzed to evaluate the learning and memory ability of the experimental animals in the reference memory test. Figure 2 illustrates the swimming patterns of the DG group, which indicates a uniformly distributed swimming trajectory across all four quadrants, with short residence times and no prolonged stays in the target quadrant. In contrast, the NOR, DCC, MgCC, and HEA groups all showed longer retention times in the target quadrant, which was significantly increased compared to the DG and UCC group (*p* < 0.05). Figure 3 shows a marked increase in platform searching time for short-term memory in the DG group when compared to the NOR group, indicating poorer memory performance in the DG group (*p* < 0.05). However, all groups administered with the test substance showed a significant decrease in the time required to reach a platform in short-term memory. No significant difference was observed between the NOR group and the other groups based on statistical analysis (*p* < 0.05). Additionally, the passive avoidance test was used as an evaluation indicator of memory learning. As depicted in Figure 4, the DCC and HEA groups showed significant improvement in the memory and learning abilities of rats (*p* < 0.05) and performed better than the UCC group.

### 3.2. Effects of C. cicadae Fermentation Products on the Expression of Inflammatory Factor Proteins in the Cortex of DG-Induced Aging Rats

Excessive intake of D-galactose can disrupt glycolysis, leading to increased osmotic pressure and impaired mitochondrial function. This, in turn, promotes the generation of free radicals and reactive oxygen species (ROS), resulting in oxidative stress [12]. Oxidative stress can trigger the release of inflammatory factors, including TNF-α, which is found in various cell types [13]. The inflammatory pathway activation can facilitate the nuclear entry of NF-κB, activating transcription factors that release additional cytokines such as iNOS and COX-2, further exacerbating the inflammatory response [14,15]. COX-2 expression has been shown to increase in the tissues of many age-related human diseases and mice, indicating that it plays a role in the aging pathway [16]. Additionally, the formation of peroxynitrite through the reaction of nitric oxide and superoxide can further exacerbate cellular damage and affect signaling pathways [17].

**Figure 5:**
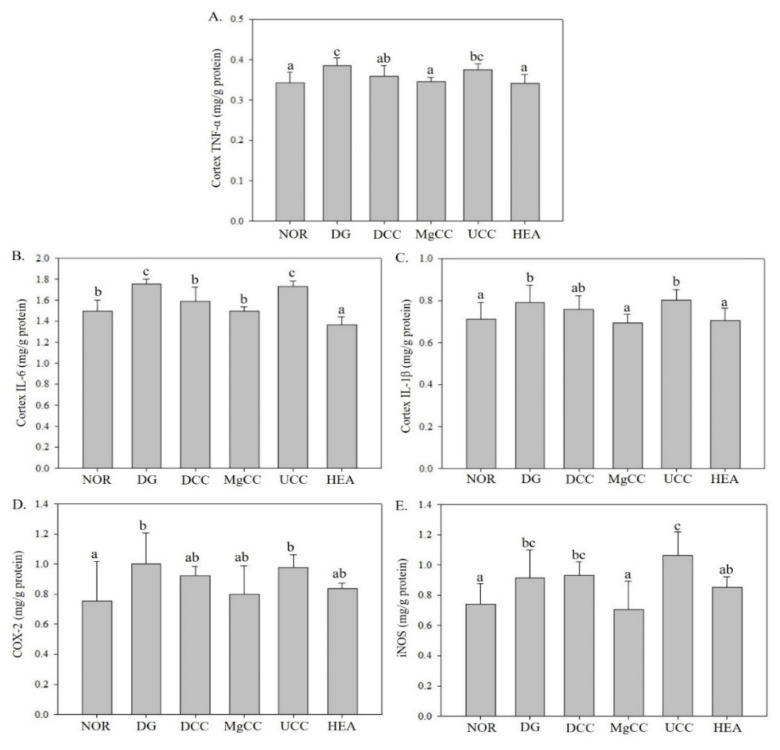
Effects of *C. cicadae* NTTU 868 fermentation products cultured with various water on the protein expressions of inflammatory factors TNF-α, IL-6, and IL-1β in the cerebral cortex of DG-induced aging rats (**A**) TNF-α, (**B**) IL-6, (**C**) IL-1β, (**D**) COX-2, (**E**) iNOS. Animals, with the exception of the NOR group, were injected daily with D-galactose (500 mg/kg b.w./day). The DCC group received daily administration of *C. cicadae* fermentation product cultured with DOW (26 mL/kg b.w./day), while the MgCC group received *C. cicadae* fermentation product cultured with MgCl_2_ solution (26 mL/kg b.w./day). The UCC group was administered *C. cicadae* fermentation product cultured with UPW (26 mL/kg b.w./day), while the HEA group was administered HEA (0.298 µL/kg b.w./day) daily. The data are presented as mean ± standard deviation (n = 7). Mean values within each group with different lowercase letters are significantly different (*p* < 0.05).

The expression levels of TNF-α, IL-1β, and IL-6 in the aging DG group induced by D-galactose were significantly higher than those in the NOR group (*p* < 0.05), as shown in Figure 5. However, the UCC group had a weaker ability to downregulate TNF-α, and the DCC group showed an intermediate level of TNF-α expression. The MgCC and HEA groups even demonstrated a significant reduction in TNF-α expression than the UCC group (*p* < 0.05), similar to the NOR group. The protein expression of TNF-α was significantly downregulated in DCC compared to DG (*p* < 0.05). The DCC, MgCC, and HEA groups showed a significant decrease in IL-6 protein expression, and the protein concentration was lower than that of the NOR group (*p* < 0.05). The expression of IL-1β protein was significantly reduced in both the MgCC and HEA groups (*p* < 0.05), reaching a level comparable to that of the NOR group. Although DCC showed a downward trend compared with DG, MgCC and HEA performed better. The results suggested that the functional component HEA in C. ci-cadae had an anti-inflammatory effect. The COX-2 expression in the DG group increased, while the MgCC and HEA groups had the downregulation, but without significant difference (*p* > 0.05). The MgCC and HEA groups showed a decreasing trend in iNOS protein content, and their effects were significantly better than those of the UCC group (*p* < 0.05). Therefore, the expression levels of TNF-α, IL-1β, IL-6, COX-2, and iNOS proteins were enhanced by d-galactose, with the MgCC and HEA groups showing greater effectiveness than the UCC group.

### 3.3. Effects of C. cicadae Fermentation Products on the Activity of Antioxidant Enzymes in the Cortex of DG-Induced Aging Rats 

Numerous studies have confirmed the theory of free radical aging, which suggests that the level of oxidative stress increases with age. This increased oxidative stress can lead to negative outcomes such as inflammation, apoptosis, and mitochondrial dysfunction. One of the main causes of this oxidative stress is the lack of antioxidant enzymes, which can neutralize peroxides and prevent cellular damage and inflammation [18].

**Figure 6:**
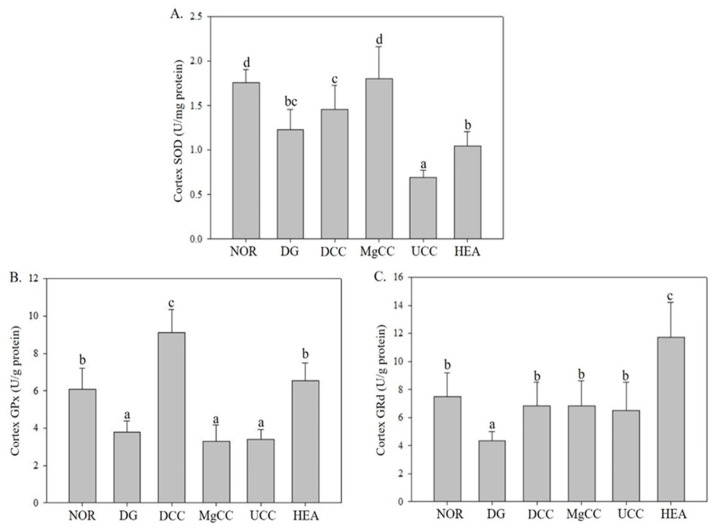
Effects of *C. cicadae* NTTU 868 fermentation products cultured with various water on the activities of GPx and GRd proteins in the cortex of DG-induced aging rats (**A**) SOD, (**B**) GPx, (**C**) GRd. The expression levels of the target proteins were determined using ELISA. Animals, with the exception of the NOR group, were injected daily with D-galactose (500 mg/kg b.w./day). The DCC group received daily administration of *C. cicadae* fermentation product cultured with DOW (26 mL/kg b.w./day), while the MgCC group received *C. cicadae* fermentation product cultured with MgCl_2_ solution (26 mL/kg b.w./day). The UCC group was administered *C. cicadae* fermentation product cultured with UPW (26 mL/kg b.w./day), while the HEA group was administered HEA (0.298 µL/kg b.w./day) daily. The data are presented as mean ± standard deviation (n = 7). Mean values within each group with different lowercase letters are significantly different (*p* < 0.05).

The effects of liquid fermentation products of *C. cicadae* on the activities of SOD, GRd, and GPx in the cerebral cortex were investigated. In Figure 6, the DG group showed significantly lower activities of SOD, GRd, and GPx than the NOR group (*p* < 0.05). The MgCC group had the highest SOD activity, followed by the DCC group. The UCC and HEA groups could not increase SOD activity. However, both the DCC and HEA groups significantly increased the GPx activity that was decreased by d-galactose. All test substance treatment groups showed an increase in GRd activity, with the HEA group showing the most significant increase. Therefore, the test substances of each group showed different effects on the regulation of antioxidant enzyme activities, but overall, the DCC, MgCC and HEA groups helped to enhance the activity of the antioxidant enzyme system, while the UCC group had a poorer effect.

### 3.4. Effects of Fermentation Products of C. cicadae on Expression of Presenilin 1 and GFAP Proteins in Cortex of DG-Induced Aging Rats

GFAP is an IF-III protein that maintains the cytoskeletal structure of glial cells and supports neighboring neurons while preserving the blood–brain barrier’s integrity. GFAP also plays a crucial role in mediating astrocyte activation following nervous system injury. Activated astrocytes alter the expression of growth factors and cytokines, causing alterations in local cellular structure and organization, ultimately leading to neuronal damage [19]. Abnormal deposition of Aβ in the brain can result from mutations in the presenilin 1 (PS1) gene on chromosome 14 or the presenilin 2 (PS2) gene on chromosome 1. These mutations lead to increased expression of Aβ protein and are associated with early-onset familial Alzheimer’s disease [20].

**Figure 7:**
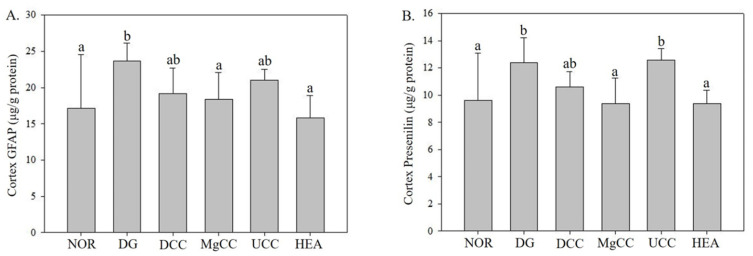
Effects of *C. cicadae* NTTU 868 fermentation products cultured with various water on the expressions of GFAP and presenilin 1 proteins in the cortex of DG-induced aging rats (**A**) GFAP (**B**) presenilin 1. The expression levels of the target protein were determined using ELISA. Animals, with the exception of the NOR group, were injected daily with D-galactose (500 mg/kg b.w./day). The DCC group received daily administration of *C. cicadae* fermentation product cultured with DOW (26 mL/kg b.w./day), while the MgCC group received *C. cicadae* fermentation product cultured with MgCl_2_ solution (26 mL/kg b.w./day). The UCC group was administered *C. cicadae* fermentation product cultured with UPW (26 mL/kg b.w./day), while the HEA group was administered HEA (0.298 µL/kg b.w./day) daily. The data are presented as mean ± standard deviation (n = 7). Mean values within each group with different lowercase letters are significantly different (*p* < 0.05).

Figure 7 shows the impact of liquid fermentation products from *C. cicadae* on GFAP expression in the cerebral cortex. GFAP expression significantly increased in the DG group (*p* < 0.05). However, the MgCC and HEA groups exhibited a significant reduction in GFAP protein expression compared to the DG group (*p* < 0.05), reaching levels comparable to the NOR group. The DCC and UCC groups had intermediate levels of GFAP expression between the NOR and DG groups. These findings suggest that fermentation products of *C. cicadae* possess neuroprotective properties and can reduce GFAP expression.

In Figure 7, the effect of liquid fermentation products of *C. cicadae* on the expression of presenilin 1 in the cerebral cortex is shown. The induced-aging DG group had significantly increased presenilin 1 protein expression (*p* < 0.05). However, the MgCC and HEA groups significantly reduced the protein expression of presenilin 1 (*p* < 0.05) to levels similar to the NOR group. The DCC group demonstrated a trend towards decreased presenilin 1 expression compared to the DG group. These results indicate that DCC can lower presenilin 1 protein expression, which may help prevent Alzheimer’s disease.

## 4. Discussion

The prolonged excessive intake of sugar can lead to oxidative stress, which, if not effectively mitigated, can result in inflammation and abnormal mitochondrial function. In turn, both inflammation and mitochondrial dysfunction can cause nerve cell apoptosis, leading to cognitive impairment [2]. Additionally, excessive intake of DG (a sugar alcohol) has been shown to increase the expression of proteins related to inflammation, such as COX-2, iNOS, NOS-2, IL-1β, IL-6, TNF-α, and NF-κB, in the brains of Kunming mice or Sprague Dawley rats. These effects were observed after the administration of DG at doses ranging from 100 to 500 mg/kg/day for 6 to 8 weeks. Moreover, the DG-treated group exhibited higher levels of reactive oxygen species (ROS) and malondialdehyde (MDA), as well as reduced antioxidant enzymes (e.g., SOD, CAT, and GSH) and total antioxidant capacity. Consequently, the synthesis of respiratory chain enzymes and ATP decreased, and mitochondrial DNA was mutated, leading to structural damage [21].

The current study revealed that the negative control group treated with D-galactose (DG) exhibited less exploration time in the target quadrant and wandering paths than the NOR group during the spatial exploration test, indicating impaired memory and learning ability. Moreover, the negative control DG group showed a significant increase in the time to find a plateau compared to the NOR group during the working memory test, which evaluates short-term memory. Additionally, the activities of antioxidant enzymes such as SOD, GRd, and GPx were found to be decreased, while the levels of inflammatory factors, including TNF-α, IL-6, and IL-1β, showed an upward trend. The inflammatory response stimulated the production of iNOS and COX-2 oxidizing factors. Furthermore, the expression levels of aging factors GFAP and Presenilin 1 were also significantly increased after the administration of D-galactose. Overall, excessive intake of D-galactose can diminish antioxidant enzyme activity, promote inflammation, and accelerate the aging process.

Prior studies have demonstrated that *C. cicadae* ethanolic extract enhances cell survival in glutamate-induced PC12 cell senescence experiments. This is achieved by reducing intracellular superoxide free radicals, inhibiting lactate dehydrogenase release, and increasing GSH-Px and SOD activity. *C. cicadae* have been shown to possess neuroprotective and antiaging properties [22]. Additionally, *C. cicadae* exhibit in vitro antioxidant activity by scavenging DPPH and superoxide anion (O_2_^−^) free radicals, thereby possessing antioxidant capacity and antiaging potential. In vitro studies also reveal that the polysaccharide of *C. cicadae* significantly enhances cell survival and reduces intracellular ROS generation in glutamic acid-induced oxidative damage in PC12 cells [23]. Previous research indicates that HEA scavenges ROS to protect PC12 cells from oxidative damage caused by H_2_O_2_, primarily by enhancing GSH-Px and SOD performance, highlighting its antioxidant capacity [8]. Our study demonstrates significant improvement in the activities of GPx and GRd in both DCC and HEA groups, with the MgCC group displaying better effects on SOD and GRd activity. Conversely, the UCC group exhibited no significant antioxidant capacity. It is hypothesized that CC fermented with DOW can enhance the regulation of biological efficacy components.

Studies have demonstrated that TNF-α upregulates the expression of IL-6 in CCl4-induced liver fibrosis. Treatment with *C. cicadae* NTTU 868 mycelium and its bioactive components, HEA and *C. cicadae* NTTU 868 mycelium, inhibits the TNF-α/IL-6 pathway [24]. In previous studies, magnesium sulfate at concentrations of 5 and 10 mmol/L significantly inhibited nitric oxide expression in LPS-activated microglia, as well as the expression of prostaglandin E2, IL-1β, TNF-α, and inducible nitric oxide synthase mRNA [25]. *C. cicadae* fermented with DOW can reduce the production of inflammatory factors in the cerebral cortex, downregulate TNF-α expression, and prevent inflammation-induced apoptosis and nerve damage. MgCC and HEA had a more pronounced effect, followed by the DCC group. *C. cicadae* and HEA have well-known anti-inflammatory properties, and magnesium ions are among the ions that effectively inhibit the production of inflammatory factors. Following an inflammatory response, additional cytokines, such as iNOS, COX-2, etc., are induced. The MgCC and HEA groups demonstrated significantly downregulated expression of iNOS, while COX-2 expression was downregulated in the DCC, MgCC, and HEA groups. Effective regulation of inflammatory factors indeed reduces the generation of subsequent oxidative factors.

Previous studies have shown that mutations in presenilin 1 lead to abnormal γ-secretase activity and increased deposition of Aβ42, which is a causative protein in Alzheimer’s disease [26]. *C. cicadae* and HEA fermented with DOW can reduce the production of GFAP and presenilin 1 in the cerebral cortex, slowing brain and nerve damage caused by aging factors and reducing the occurrence of senile diseases. MgCC showed superior regulation ability. Magnesium ions have been shown to promote synaptic plasticity and improve learning and memory [27], and they are effective in inhibiting aging-related factors. *C. cicadae* have neuroprotective properties and slow the production of aging-related factors. 

This study investigates how minerals in DOW enhance the antiaging effects of *C. cicadae* fermentation products. Minerals in DOW such as magnesium, calcium, selenium, manganese, and others have important roles in human health and disease prevention. In recent years, more and more studies have shown that minerals in DOW can regulate cellular metabolism, antioxidant stress, inflammation inhibition, and neural function improvement among other physiological processes. Magnesium, calcium, selenium, and manganese are essential minerals for the human body, and they play important roles in the function and health of the nervous system. Magnesium is an important ion that participates in regulating neurotransmission, energy metabolism, antioxidant, and anti-inflammatory processes. Magnesium deficiency can cause neuronal damage and death, increasing the risk of neurodegenerative diseases such as Alzheimer’s disease. Magnesium supplementation may be beneficial for protecting neurons, reducing chronic inflammation, and improving cognitive function [28]. Calcium is an important signaling molecule that participates in regulating neuronal excitability, neurotransmitter release, activating signal transduction, and other processes. Calcium imbalance can cause neuronal apoptosis or necrosis, promoting protein aggregation and oxidative stress, increasing the risk of neurodegenerative diseases such as Parkinson’s disease. Calcium supplementation may be beneficial for maintaining the normal neuronal function, inhibiting neurotoxicity, and improving learning and memory [29]. Selenium is a powerful antioxidant that participates in synthesizing selenoproteins and glutathione and other molecules, protecting neurons from free radical attacks. Selenium deficiency or excess can both cause nervous system damage, increasing the risk of dementia and other diseases. Selenium supplementation may be beneficial for enhancing antioxidant capacity, reducing protein aggregation, and improving cognitive function [30,31]. Manganese is a trace element that has important functions for the nervous system under normal conditions, such as synthesizing dopamine and other neurotransmitters, regulating energy metabolism and so on. However, manganese excess can cause manganese poisoning, resulting in neurodegenerative changes such as Parkinsonian syndrome. Manganese poisoning affects protein folding, cytochrome c oxidase activity, neurotransmitter metabolisms, and other processes, leading to neuronal damage and death. Manganese supplementation may have some help in preventing or treating some neurodegenerative diseases, but more research and evidence are needed to support this hypothesis. 

In a previous study of DOW, minerals in DOW such as magnesium and calcium can promote mitochondrial biogenesis, increasing mitochondrial DNA (mtDNA) content and cytochrome c oxidase activity through the AMPK activation signaling pathway [32]. Mitochondria are key components of cellular energy metabolism and apoptosis, and their dysfunction is related to neurodegenerative diseases. Our previous studies have suggested that the level of magnesium ions in the brain is related to the formation of amyloid and phosphorylated tau proteins [9]. This study used minerals in DOW to cultivate *C. cicadae* mycelium, and found that these minerals not only promoted mycelial growth, but also were absorbed by mycelia and increased bioavailability. This study compared the fermentation products of *C. cicadae* cultivated with magnesium chloride and ultrapure water, and found that the former had better antioxidant and anti-inflammatory effects. Therefore, this study speculated that organic magnesium might help aging animals absorb more magnesium ions, thereby improving brain aging. In addition, this study also confirmed that minerals in DOW could be absorbed through *C. cicadae* fermentation products, and had a positive effect on alleviating brain damage and aging caused by Aβ40 [9].

**Figure 8:**
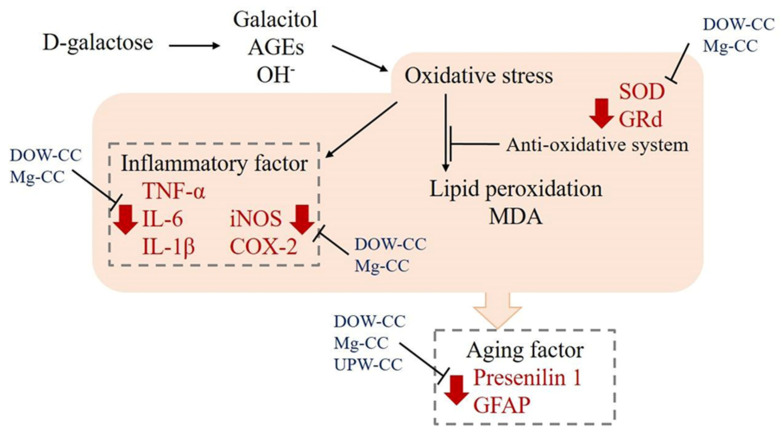
Effect of water source on fermentation products of *C. cicadae* NTTU 868 and their impact on oxidation and inflammation in the brains of D-galactose-induced aging mice.

In conclusion, DOW-fermented *C. cicadae* and HEA were found to increase SOD and GRd activity, scavenge free radicals, and reduce the inflammatory response caused by oxidative stress (Figure 8). MgCC had superior efficacy. MgCC also inhibited TNF-α to reduce the expression of downstream inflammatory response mediators iNOS and COX-2, and had a regulatory effect on the expression of aging-related factors GFAP and presenilin 1. These findings demonstrate that the regulation of the expression of anti-inflammatory, antioxidant, and aging-related factors of *C. cicadae* products fermented with DOW can prevent aging and show significant improvement in efficacy.

## Data Availability

All data included in this study are available upon request by contacting the corresponding authors.

## References

[1] Harraan D. (2002). Aging: A theory based on free radical and radiation chemistry. Sci. Aging Knowl. Environ..

[2] Shwe T., Pratchayasakul W., Chattipakorn N., Chattipakorn S.C. (2018). Role of D-galactose-induced brain aging and its potential used for therapeutic interventions. Exp. Gerontol..

[3] Hung Y.-P., Lee C.-L. (2017). Higher anti-liver fibrosis effect of *Cordyceps militaris*-fermented product cultured with deep ocean water via inhibiting proinflammatory factors and fibrosis-related factors expressions. Mar. Drugs.

[4] Hsu C.-L., Chang Y.-Y., Chiu C.-H., Yang K.-T., Wang Y., Fu S.-G., Chen Y.-C. (2011). Cardiovascular protection of deep-seawater drinking water in high-fat/cholesterol fed hamsters. Food Chem..

[5] Wang L.-C., Lung T.-Y., Kung Y.-H., Wang J.-J., Tsai T.-Y., Wei B.-L., Pan T.-M., Lee C.-L. (2013). Enhanced anti-obesity activities of red mold dioscorea when fermented using deep ocean water as the culture water. Mar. Drugs.

[6] Wang L.-C., Kuo I.-U., Tsai T.-Y., Lee C.-L. (2013). *Antrodia camphorata*-fermented product cultured in deep ocean water has more liver protection against thioacetamide-induced fibrosis. Appl. Microbiol. Biotechnol..

[7] Lu M.-Y., Chen C.-C., Lee L.-Y., Lin T.-W., Kuo C.-F. (2015). N 6-(2-Hydroxyethyl) adenosine in the medicinal mushroom *Cordyceps cicadae* attenuates lipopolysaccharide-stimulated pro-inflammatory responses by suppressing TLR4-mediated NF-κB signaling pathways. J. Nat. Prod..

[8] Zhang L., Wu T., Olatunji O.J., Tang J., Wei Y., Ouyang Z. (2019). N 6-(2-hydroxyethyl)-adenosine from *Cordyceps cicadae* attenuates hydrogen peroxide induced oxidative toxicity in PC12 cells. Metab. Brain Dis..

[9] Wu Y.-Z., Lee C.-L. (2021). *Cordyceps cicadae* NTTU 868 Mycelium with The Addition of Bioavailable Forms of Magnesium from Deep Ocean Water Prevents the Aβ40 and Streptozotocin-Induced Memory Deficit via Suppressing Alzheimer’s Disease Risk Factors and Increasing Magnesium Uptake of Brain. Fermentation.

[10] Morris R.G., Garrud P., Rawlins J.A., O’Keefe J. (1982). Place navigation impaired in rats with hippocampal lesions. Nature.

[11] Suits E., Isaacson R.L. (1968). The effects of scopolamine hydrobromide on one-way and two-way avoidance learning in rats. Int. J. Neuropharmacol..

[12] Qu Z., Zhang J., Yang H., Huo L., Gao J., Chen H., Gao W. (2016). Protective effect of tetrahydropalmatine against d-galactose induced memory impairment in rat. Physiol. Behav..

[13] de Gonzalo-Calvo D., Neitzert K., Fernández M., Vega-Naredo I., Caballero B., García-Macía M., Suárez F.M., Rodríguez-Colunga M.J., Solano J.J., Coto-Montes A. (2010). Differential inflammatory responses in aging and disease: TNF-α and IL-6 as possible biomarkers. Free Radic. Biol. Med..

[14] Chen Y.-F., Wang Y.-W., Huang W.-S., Lee M.-M., Wood W.G., Leung Y.-M., Tsai H.-Y. (2016). Trans-cinnamaldehyde, an essential oil in cinnamon powder, ameliorates cerebral ischemia-induced brain injury via inhibition of neuroinflammation through attenuation of iNOS, COX-2 expression and NFκ-B signaling pathway. Neuromol. Med..

[15] Lawrence T., Gilroy D.W., Colville-Nash P.R., Willoughby D.A. (2001). Possible new role for NF-κB in the resolution of inflammation. Nat. Med..

[16] Andreasson K.I., Savonenko A., Vidensky S., Goellner J.J., Zhang Y., Shaffer A., Kaufmann W.E., Worley P.F., Isakson P., Markowska A.L. (2001). Age-dependent cognitive deficits and neuronal apoptosis in cyclooxygenase-2 transgenic mice. J. Neurosci..

[17] Griendling K.K., FitzGerald G.A. (2003). Oxidative stress and cardiovascular injury: Part I: Basic mechanisms and in vivo monitoring of ROS. Circulation.

[18] Grune T. (2000). Oxidative stress, aging and the proteasomal system. Biogerontology.

[19] Bernal G.M., Peterson D.A. (2011). Phenotypic and gene expression modification with normal brain aging in GFAP-positive astrocytes and neural stem cells. Aging Cell.

[20] Head E., Torp R. (2002). Insights into Aβ and presenilin from a canine model of human brain aging. Neurobiol. Dis..

[21] Azman K.F., Zakaria R. (2019). D-Galactose-induced accelerated aging model: An overview. Biogerontology.

[22] Olatunji O.J., Feng Y., Olatunji O.O., Tang J., Ouyang Z., Su Z., Wang D., Yu X. (2016). Neuroprotective effects of adenosine isolated from *Cordyceps cicadae* against oxidative and ER stress damages induced by glutamate in PC12 cells. Environ. Toxicol. Pharmacol..

[23] Olatunji O.J., Feng Y., Olatunji O.O., Tang J., Wei Y., Ouyang Z., Su Z. (2016). Polysaccharides purified from *Cordyceps cicadae* protects PC12 cells against glutamate-induced oxidative damage. Carbohydr. Polym..

[24] Ke B.-J., Lee C.-L. (2018). *Cordyceps cicadae* NTTU 868 mycelium prevents CCl4-induced hepatic fibrosis in BALB/c mice via inhibiting the expression of pro-inflammatory and pro-fibrotic cytokines. J. Funct. Foods.

[25] Gao F., Ding B., Zhou L., Gao X., Guo H., Xu H. (2013). Magnesium sulfate provides neuroprotection in lipopolysaccharide-activated primary microglia by inhibiting NF-κB pathway. J. Surg. Res..

[26] Wolfe M.S., Xia W., Ostaszewski B.L., Diehl T.S., Kimberly W.T., Selkoe D.J. (1999). Two transmembrane aspartates in presenilin-1 required for presenilin endoproteolysis and γ-secretase activity. Nature.

[27] Billard J.M., Vink R., Nechifor M. (2011). Brain free magnesium homeostasis as a target for reducing cognitive aging. Magnesium in the Central Nervous System.

[28] Maier J.A.M., Locatelli L., Fedele G., Cazzaniga A., Mazur A. (2022). Magnesium and the Brain: A Focus on Neuroinflammation and Neurodegeneration. Int. J. Mol. Sci..

[29] Mattson M.P. (2007). Calcium and neurodegeneration. Aging Cell.

[30] Cai Z., Zhang J., Li H. (2019). Selenium, aging and aging-related diseases. Aging Clin. Exp. Res..

[31] Zhao Y., Jia M., Chen W., Liu Z. (2022). The neuroprotective effects of intermittent fasting on brain aging and neurodegenerative diseases via regulating mitochondrial function. Free Radic. Biol. Med..

[32] Sheu M.J., Chou P.Y., Lin W.H., Pan C.H., Chien Y.C., Chung Y.L., Liu F.C., Wu C.H. (2013). Deep sea water modulates blood pressure and exhibits hypolipidemic effects via the AMPK-ACC pathway: An in vivo study. Mar. Drugs.

